# Eosinophilic granulomatosis with polyangiitis complicated by pulmonary aspergillosis and misdiagnosed as allergic bronchopulmonary aspergillosis: a case report

**DOI:** 10.3389/fmed.2026.1894051

**Published:** 2026-07-24

**Authors:** Hongmei Sheng, Wei Zhang, Jun Lv, Qihong Yu

**Affiliations:** Department of Respiratory and Critical Care Medicine, Tianjin Chest Hospital, Tianjin, China

**Keywords:** allergic bronchopulmonary aspergillosis, asthma, diagnosis, differential, eosinophilic granulomatosis with polyangiitis, mepolizumab

## Abstract

**Objectives:**

To enhance the diagnostic and therapeutic awareness of eosinophilic granulomatosis with polyangiitis (EGPA) complicated by pulmonary aspergillosis.

**Methods:**

We report a case of EGPA initially misdiagnosed as allergic bronchopulmonary aspergillosis (ABPA) in a 52-year-old female patient.

**Results:**

The patient presented with intermittent wheezing for more than six months and had a history of sinusitis. An outside hospital diagnosed ABPA based on pulmonary opacities, bronchiectasis, and evidence of *Aspergillus* infection, but standard therapy proved ineffective. Upon admission, laboratory findings revealed a markedly elevated absolute peripheral blood eosinophil count (5.29 × 10^9^/L) and positivity for MPO-ANCA and p-ANCA. Serum total IgE was 8.85 IU/mL, and specific IgE to *Aspergillus fumigatus* was <0.10 IU/mL, both of which were inconsistent with ABPA. Chest CT showed multiple bilateral patchy and nodular opacities with bronchiectasis. Bronchoalveolar lavage fluid targeted next-generation sequencing (tNGS) detected *Aspergillus* at the genus level (23 sequence reads, relative abundance 33.39%). The diagnosis was revised to EGPA complicated by pulmonary aspergillosis. The patient was treated with glucocorticoids combined with mepolizumab, supplemented with voriconazole. Following treatment, the patient’s symptoms resolved, with near normalization of imaging findings and pulmonary function. After 10 months of follow-up, methylprednisolone was completely discontinued in December 2025, and at the last follow-up in May 2026, the patient had been off glucocorticoids for 5 months, with sustained remission and successful extension of the mepolizumab dosing interval to 8 weeks.

**Conclusion:**

For patients presenting with refractory asthma accompanied by eosinophilia and pulmonary opacities, EGPA should be highly suspected. Glucocorticoids combined with mepolizumab is effective. In patients achieving sustained remission, extending the mepolizumab dosing interval to 8 weeks may be a safe and effective long-term maintenance strategy in carefully selected patients, though this observation requires further validation in prospective studies.

## 1 Introduction

Eosinophilic granulomatosis with polyangiitis (EGPA), formerly known as Churg-Strauss syndrome, is a rare, multisystem necrotizing vasculitis characterized by asthma, eosinophilia, and granulomatous inflammation (1). The diagnosis of EGPA can be challenging, particularly when it mimics other conditions such as allergic bronchopulmonary aspergillosis (ABPA), which shares overlapping clinical features including asthma, eosinophilia, and pulmonary infiltrates (2). However, the treatment strategies and prognoses for these two conditions differ substantially. While the diagnostic overlap between EGPA and ABPA is a well-recognized clinical challenge, our case offers three distinct novel aspects: (1) successful extension of the mepolizumab dosing interval to 8 weeks with sustained remission; (2) complete glucocorticoid discontinuation for 5 months; and (3) the use of total IgE and specific IgE to *Aspergillus* as key diagnostic evidence to differentiate EGPA from ABPA in a patient with concurrent *Aspergillus* colonization detected by BALF tNGS. Here, we report this case to provide valuable clinical insights for differential diagnosis, treatment optimization, and long-term management of EGPA complicated by pulmonary aspergillosis.

## 2 Case presentation

### 2.1 Patient information and history

A 52-year-old female patient was admitted to our hospital on 5 July 2025, with a chief complaint of “intermittent wheezing for more than 6 months.” Six months prior to admission, the patient developed chest tightness, wheezing, and white sputum production without obvious precipitating factors. An outside hospital diagnosed “bronchial asthma” and provided symptomatic treatment with temporary relief. One month prior to admission, the patient experienced worsening wheezing. Chest CT at the outside hospital revealed “bronchiectasis with infection,” and bronchoalveolar lavage fluid next-generation sequencing (NGS) detected *Aspergillus*. The patient was diagnosed with ABPA and treated with voriconazole, micafungin, methylprednisolone, and piperacillin-tazobactam for one week, with no significant improvement. One week after discharge, wheezing recurred, prompting her visit to our outpatient clinic. The patient had a past medical history of sinusitis.

### 2.2 Diagnostic assessment

Physical examination: Vital signs were stable. Breath sounds were coarse bilaterally with scattered dry rales. No rashes, clubbing, or neurological abnormalities were noted.

Laboratory investigations: Blood routine showed white blood cells 12.2 × 10^9^/L and absolute eosinophil count 5.29 × 10^9^/L (43.4%). Autoantibody testing was positive for MPO-ANCA and p-ANCA. Serum total IgE was 8.85 IU/mL, and specific IgE to *Aspergillus fumigatus* was <0.10 IU/mL.

**FIGURE 1:**
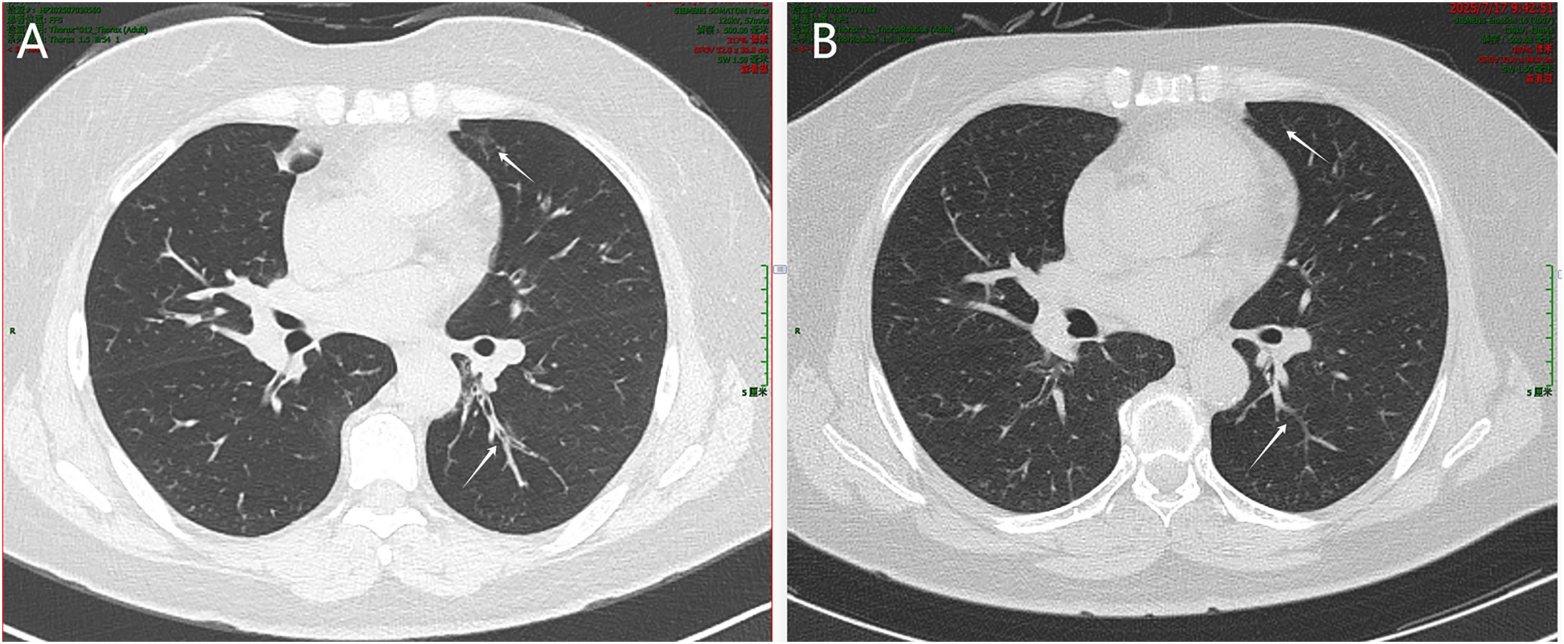
Comparison of chest CT findings before and after treatment. **(A)** Pretreatment chest CT (lung window, 2025-07-03) shows multiple bilateral patchy and nodular opacities with bronchiectasis (arrows). **(B)** Post-treatment chest CT (lung window, 2025-07-17) shows significant resolution of the pulmonary lesions and improvement of bronchiectasis (arrows).

Imaging: Chest CT (3 July 2025) showed multiple bilateral patchy and nodular opacities with bronchiectasis (Figure 1A).

Pulmonary function tests (pre-treatment): Revealed severe mixed ventilatory dysfunction (FEV1/FVC 54.21%, FEV1 39.2% of predicted value), negative bronchodilator test (FEV1 improvement 10.53%), and fractional exhaled nitric oxide (FeNO) of 98 ppb.

Bronchoscopy and pathogen testing: Bronchoscopy revealed diffuse purulent secretions in the bilateral bronchi, predominantly in the lingular segment of the left upper lobe and the dorsal segment of the left lower lobe. Bronchoalveolar lavage fluid was sent for targeted next-generation sequencing (tNGS), which qualitatively detected *Aspergillus* at the genus level (23 sequence reads, relative abundance 33.39%). No other significant bacterial or viral pathogens were identified. The specific *Aspergillus* species was not further differentiated by the assay, which we acknowledge as a limitation.

Final diagnosis: Based on the presence of asthma, marked eosinophilia (>10%), sinusitis, non-fixed pulmonary infiltrates, and ANCA positivity, the patient met the diagnostic criteria for EGPA. The extremely low total IgE and negative specific IgE to *Aspergillus fumigatus* effectively excluded ABPA, further supporting the diagnosis of EGPA. The final diagnosis was EGPA complicated by pulmonary aspergillosis.

### 2.3 Therapeutic intervention and follow-up

The patient was initially treated with intravenous methylprednisolone 40 mg/day combined with oral voriconazole (200 mg twice daily for a total of 6 weeks) to control inflammation and infection. After initial stabilization, to facilitate glucocorticoid tapering and long-term disease control, mepolizumab 300 mg subcutaneously every 4 weeks was added starting from the second week of treatment. The first six cycles were administered at this 4-week interval. Subsequently, due to sustained disease stability, the dosing interval was extended to every 8 weeks.

**FIGURE 2:**
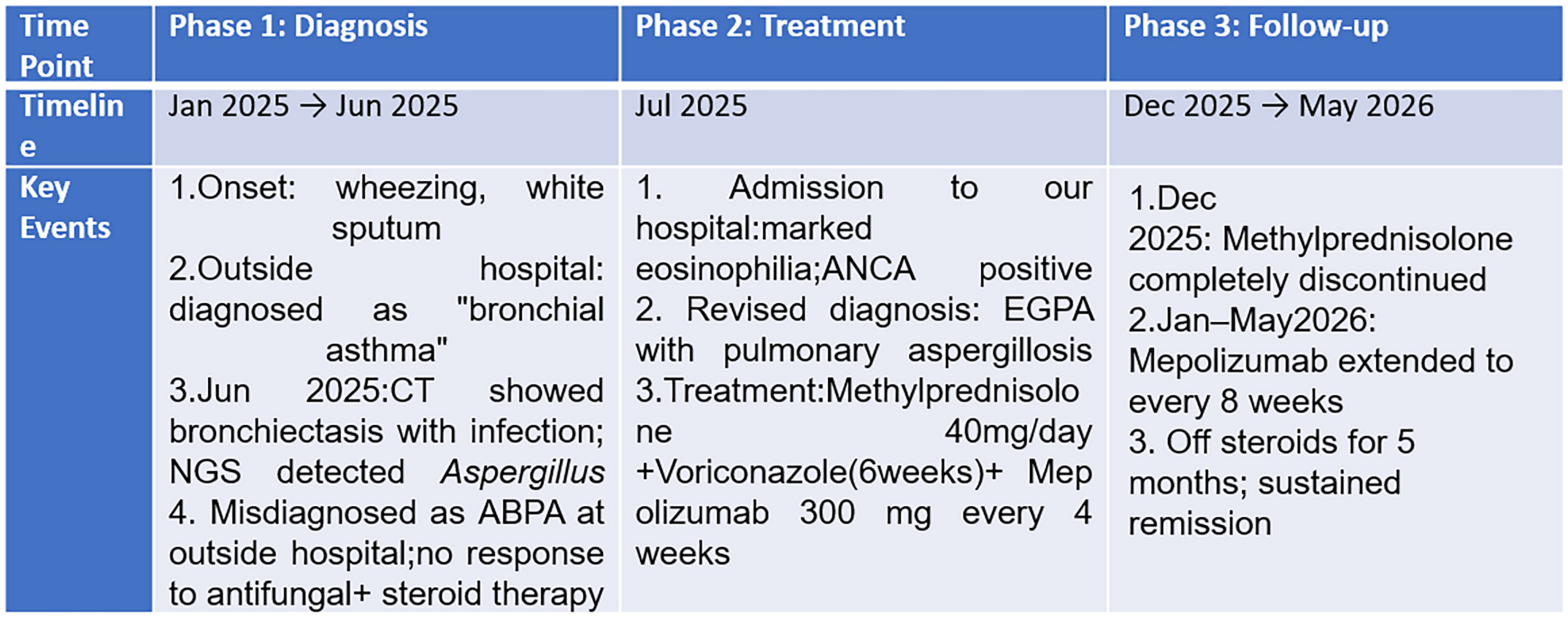
Timeline of diagnosis and treatment. The timeline illustrates the complete clinical course from symptom onset, misdiagnosis at an outside hospital, correct diagnosis at our center, induction therapy with mepolizumab 300 mg every 4 weeks plus glucocorticoids and voriconazole, to long-term follow-up (10 months) during which the mepolizumab interval was successfully extended to 8 weeks and methylprednisolone was completely discontinued in December 2025 (off steroids for 5 months at the last follow-up).

After 10 days of treatment, repeat chest CT showed significant absorption and reduction of pulmonary lesions (Figure 1B), and pulmonary function improved. Following completion of voriconazole, mepolizumab was continued. The patient was followed regularly for 10 months (until May 2026). Wheezing completely resolved, and glucocorticoids were successfully tapered stepwise: methylprednisolone was gradually reduced from 40 mg/day and was completely discontinued in December 2025. At the last follow-up in May 2026, the patient had been off methylprednisolone for 5 months, with no signs of relapse. At the last follow-up, peripheral blood eosinophil counts remained normal, pulmonary function remained normal (FEV1 94% of predicted value), and FeNO remained below 15 ppb. The patient remained asymptomatic with no cough or wheezing and was in good general condition. The patient remains in sustained remission with good tolerability. The complete diagnostic and therapeutic process is summarized in Figure 2.

## 3 Discussion

### 3.1 Etiology and clinical spectrum of EGPA

The etiology of EGPA remains incompletely understood but is currently thought to involve immune dysregulation triggered by environmental factors in genetically susceptible individuals. The pathogenesis involves both eosinophil-mediated inflammation and ANCA-associated necrotizing vasculitis (1). These mechanisms determine the classic three-phase clinical spectrum of EGPA: prodromal phase (allergic rhinitis, asthma), eosinophilic infiltration phase (Löffler syndrome, eosinophilic pneumonia, gastroenteritis), and systemic vasculitis phase (purpura, mononeuritis multiplex, glomerulonephritis) (2).

### 3.2 Diagnostic criteria and differential diagnosis

Current EGPA diagnosis is based on the 1990 American College of Rheumatology (ACR) classification criteria, which include asthma, eosinophilia (>10%), mononeuropathy or polyneuropathy, non-fixed pulmonary infiltrates, paranasal sinus abnormalities, and extravascular eosinophilic infiltration, with four of six criteria required for diagnosis (3). In addition, the 2022 ACR/EULAR classification criteria for EGPA have been developed to improve diagnostic sensitivity and specificity (4). Our patient fulfilled both the 1990 criteria (asthma, eosinophilia > 10%, sinusitis, and non-fixed pulmonary infiltrates) and the 2022 criteria, confirming the diagnosis with high confidence. Our patient met the diagnostic criteria by presenting with asthma, marked eosinophilia, sinusitis, and pulmonary infiltrates. ANCA positivity is a key serological feature supporting EGPA and distinguishing it from ABPA.

Regarding the clinical significance of ANCA, recent studies have provided new insights. A 2025 Korean single-center cohort study showed that MPO-ANCA-positive patients exhibited higher disease activity and systemic inflammatory marker levels compared with ANCA-negative patients, but no significant differences in long-term outcomes were observed between the two groups (5). Our patient was double-positive for MPO-ANCA/p-ANCA, and her clinical course was characterized predominantly by pulmonary infiltrates and sinusitis, without severe renal or neurological involvement, suggesting that the phenotype-predictive value of ANCA in EGPA requires further validation.

### 3.3 EGPA versus ABPA: diagnostic pitfalls

Eosinophilic granulomatosis with polyangiitis and ABPA share overlapping features of asthma, eosinophilia, and pulmonary infiltrates. However, the differential diagnosis is critically informed by IgE testing. In our patient, the extremely low total IgE (8.85 IU/mL) and negative specific IgE to *Aspergillus fumigatus* (<0.10 IU/mL) effectively excluded allergic sensitization to *Aspergillus*, which is a hallmark of ABPA. Key differentiating points include: (1) ANCA: positivity for MPO-ANCA/p-ANCA strongly suggests EGPA, while ABPA is typically ANCA-negative; (2) Multisystem involvement: extrapulmonary manifestations such as sinusitis, peripheral neuropathy, and cutaneous lesions point toward EGPA; (3) Treatment response: poor response to standard ABPA therapy should prompt reconsideration of EGPA (6).

### 3.4 EGPA with aspergillosis: pathophysiological and management considerations

Eosinophilic granulomatosis with polyangiitis and ABPA share overlapping clinical features, but their underlying pathophysiological mechanisms differ. Both diseases are characterized by Th2-driven eosinophilic inflammation; however, EGPA is a systemic necrotizing vasculitis with ANCA-associated vascular injury, whereas ABPA is a hypersensitivity reaction to *Aspergillus* antigens in the airways. Current hypotheses suggest that these conditions may represent a continuum in certain phenotypes, with fungal sensitization potentially triggering or exacerbating eosinophilic inflammation in genetically susceptible individuals. The therapeutic convergence of these two entities is an area of emerging interest. Exploratory evidence suggests that mepolizumab, an anti-IL-5 monoclonal antibody, may have potential benefits in both conditions by reducing eosinophilic inflammation (7). In our patient, the presence of ANCA positivity, multisystem involvement, and the extremely low total IgE with negative specific IgE to *Aspergillus fumigatus* strongly supported the diagnosis of EGPA rather than ABPA. The excellent response to mepolizumab is consistent with its established efficacy in eosinophilic EGPA.

Structural airway damage secondary to EGPA is a risk factor for *Aspergillus* infection (6). The success of our case relied on a dual strategy: controlling acute infection with voriconazole while fundamentally improving the pulmonary environment by suppressing EGPA vasculitis activity.

### 3.5 Long-term mepolizumab therapy and dose optimization

Mepolizumab is the only anti-IL-5 monoclonal antibody approved for EGPA, with a recommended dose of 300 mg subcutaneously every 4 weeks. The MIRRA trial demonstrated its efficacy in significantly improving remission rates, reducing relapse risk, and decreasing oral glucocorticoid requirements (8). Its long-term efficacy has been further confirmed in multiple studies. The open-label extension of the MANDARA trial showed that after 2 years of anti-IL-5/5R therapy, 62%–68% of patients maintained remission, and 44% achieved complete discontinuation of oral glucocorticoids (9).

In real-world data from the Chinese population, a 2025 single-center retrospective study showed that after 12 months of mepolizumab treatment, the median daily glucocorticoid dose decreased from 10 mg to 5 mg (*P* < 0.001), and FEV1% predicted increased from 69.3% to 81.4% (*P* = 0.004), indicating good efficacy and safety of mepolizumab in Chinese patients (10).

A key clinical implication of this case is the optimization of the dosing interval and the achievement of complete glucocorticoid discontinuation. A 2026 Japanese retrospective cohort study systematically evaluated the safety and efficacy of extending the mepolizumab injection interval for the first time: among 43 EGPA patients, 10 extended their injection interval to 5–6 weeks, with a median extended interval duration of 10.5 months. The results showed no significant increase in relapse rates or blood eosinophil counts after interval extension (11). A Spanish multicenter real-world study similarly confirmed that mepolizumab enabled glucocorticoid dose reduction in 79.3% of patients and complete discontinuation in 57.1% (12).

Our case achieved even better treatment outcomes. After completing six cycles of regular 4-week dosing, based on complete remission, we extended the interval to 8 weeks. The patient has maintained this regimen for 6 months without relapse. More notably, the patient successfully discontinued methylprednisolone in December 2025 and had been off glucocorticoids for 5 months at the last follow-up in May 2026. This observation suggests that for EGPA patients achieving deep remission, the mepolizumab dosing interval may not need to be strictly maintained at 4 weeks; extension to 8 weeks may still effectively control disease activity and may enable complete glucocorticoid discontinuation in carefully selected patients. However, we acknowledge that this observation is based on a single case with a limited, non-severe phenotype (ENT, upper airway, and lung involvement, without renal or neurological manifestations). Therefore, this finding is not generalizable to all EGPA patients, and the decision to extend the dosing interval should be made cautiously on an individual basis. Prospective studies and larger cohorts are needed to validate the safety and efficacy of this dose-spacing approach. At present, this observation should be considered as hypothesis-generating rather than practice-changing. A step-down strategy (induction at the standard interval followed by individualized interval extension) can help maintain efficacy while reducing healthcare burden, minimizing glucocorticoid-related side effects, and improving quality of life.

### 3.6 Summary and clinical implications

In summary, this case offers clinical teaching and academic value in four aspects: (1) Diagnosis: clearly demonstrates the differential diagnostic pathway between EGPA and ABPA, with IgE testing playing a crucial role in excluding ABPA; (2) Management of complications: successfully manages EGPA complicated by pulmonary aspergillosis; (3) Therapy: validates the rapid induction of remission with glucocorticoids combined with mepolizumab; (4) Long-term management (unique highlight): based on existing evidence, this case is among the first to successfully extend the mepolizumab dosing interval from 4 to 8 weeks and achieve complete glucocorticoid discontinuation (off steroids for 5 months), providing a new reference for individualized, step-down treatment of EGPA in selected patients.

### 3.7 Limitations

We acknowledge several limitations. First, this is a single case report, which limits the generalizability of our findings. Second, the BALF tNGS assay detected *Aspergillus* at the genus level only; species-level identification (e.g., *Aspergillus fumigatus* vs. *Aspergillus flavus*) was not available. Third, skin prick testing was not performed. Fourth, the observation regarding 8-week mepolizumab interval extension is based on a single patient with limited, non-severe disease and requires validation in larger prospective studies. Despite these limitations, the clinical context—including the extremely low total IgE, negative specific IgE to *Aspergillus fumigatus*, and the patient’s excellent response to immunosuppressive therapy—strongly supports the diagnosis of EGPA rather than ABPA.

## 4 Conclusion

Respiratory physicians, as the first-line clinicians often encountering EGPA patients, should maintain a high index of suspicion for this disease. For patients presenting with refractory asthma, eosinophilia, and pulmonary opacities, systematic application of the ACR diagnostic criteria and routine ANCA testing are essential to avoid misdiagnosis. IgE testing (total IgE and specific IgE to *Aspergillus*) provides critical diagnostic information in the differential diagnosis of EGPA versus ABPA. This case demonstrates that glucocorticoids can rapidly induce EGPA remission, and the addition of mepolizumab facilitates glucocorticoid tapering, long-term stability, and even complete discontinuation. After sustained remission is achieved, extending the mepolizumab dosing interval to 8 weeks may be a safe and effective long-term maintenance strategy in carefully selected patients, though this observation requires further validation in prospective studies.

## Data Availability

The original contributions presented in this study are included in this article/supplementary material, further inquiries can be directed to the corresponding author.
